# Heterogeneity and Architecture of Pathological Prion Protein Assemblies: Time to Revisit the Molecular Basis of the Prion Replication Process?

**DOI:** 10.3390/v11050429

**Published:** 2019-05-10

**Authors:** Angélique Igel-Egalon, Jan Bohl, Mohammed Moudjou, Laetitia Herzog, Fabienne Reine, Human Rezaei, Vincent Béringue

**Affiliations:** 1 Molecular Virology and Immunology Unit (VIM), INRA, Université Paris-Saclay, 78350 Jouy-en-Josas, France; jan.bohl@u-psud.fr (J.B.); mohammed.moudjou@inra.fr (M.M.); laetitia.herzog@inra.fr (L.H.); fabienne.reine@inra.fr (F.R.); human.rezaei@inra.fr (H.R.); 2 Laboratory of Physical Chemistry (LCP), UMR 8000 CNRS, Université Paris Sud, 91400 Orsay, France

**Keywords:** prion, PrP, amyloid, quasi-species, dynamics

## Abstract

Prions are proteinaceous infectious agents responsible for a range of neurodegenerative diseases in animals and humans. Prion particles are assemblies formed from a misfolded, β-sheet rich, aggregation-prone isoform (PrP^Sc^) of the host-encoded cellular prion protein (PrP^C^). Prions replicate by recruiting and converting PrP^C^ into PrP^Sc^, by an autocatalytic process. PrP^Sc^ is a pleiomorphic protein as different conformations can dictate different disease phenotypes in the same host species. This is the basis of the strain phenomenon in prion diseases. Recent experimental evidence suggests further structural heterogeneity in PrP^Sc^ assemblies within specific prion populations and strains. Still, this diversity is rather seen as a size continuum of assemblies with the same core structure, while analysis of the available experimental data points to the existence of structurally distinct arrangements. The atomic structure of PrP^Sc^ has not been elucidated so far, making the prion replication process difficult to understand. All currently available models suggest that PrP^Sc^ assemblies exhibit a PrP^Sc^ subunit as core constituent, which was recently identified. This review summarizes our current knowledge on prion assembly heterogeneity down to the subunit level and will discuss its importance with regard to the current molecular principles of the prion replication process.

## 1. Introduction

Prion diseases are a group of fatal neurodegenerative disorders that include scrapie in sheep and goats, bovine spongiform encephalopathies in cattle, chronic wasting disease in cervids, and Creutzfeldt-Jakob disease in humans [1]. These diseases are all caused by prions, an infectious agent of proteinaceous nature, exclusively composed of misfolded isoforms (PrP^Sc^) of the host-encoded prion protein (PrP^C^). During the disease pathogenesis, the PrP^Sc^ seeds, either acquired through infection or elicited from spontaneous conversion of wild-type or mutated PrP^C^, template the conversion of host-PrP^C^ into PrP^Sc^ by an autocatalytic manner, resulting in the deposition of pathogenic PrP^Sc^ assemblies in the brain and sometimes in extraneural tissues, such as the lymphoid tissue [2]. The biochemical properties of PrP^Sc^ and PrP^C^ strongly differ. PrP^Sc^ is β-sheet rich, contains a protease-resistant core and is prone to aggregation (for review [3]).

In susceptible mammals, including laboratory species, PrP^Sc^ shows a remarkable ability to form structurally distinct PrP^Sc^ assemblies at the level of the tertiary and quaternary protein structure, known as prion strains. These prion strains encode unique stereotypical biological phenotypes defined by the time course to disease, neuropathological features, and tropism for specific brain regions or the lymphoid tissue [4,5,6,7]. PrP^Sc^ structural polymorphism is mostly considered as between strain polymorphism. However, experimental evidence supports the view for further structural diversity of PrP^Sc^ assemblies within specific prion populations and strains. This diversity can be revealed during adaptive prion transmission events [8,9,10,11], kinetic studies of prion pathogenesis [12,13,14] or be evidenced biochemically, as detailed in the first part of the review, notably with sedimentation velocity (SV) methods. Whether this assembly diversity represents a size continuum of particles with the same core structure or more fundamentally, structurally distinct arrangements will be discussed. 

The atomic structure of PrP^C^ has been comprehensively characterized (review [15]). In contrast to other misfolded proteins associated with neurodegenerative diseases (e.g., Alzheimer’s disease, Parkinson’s disease), physiological PrP^C^ is folded, meaning that during prion pathogenesis, PrP^C^ must unfold and refold [16]. So far, the atomic resolution structure of PrP^Sc^ and the architecture of PrP^Sc^ assemblies have not been resolved, due to the intrinsic heterogeneity of the protein samples. Such information is key for the molecular understanding of the prion replication process, including unfolding of PrP^C^, its conversion into PrP^Sc^, and the formation of supramolecular amyloidogenic assemblies. The currently available models, based on electronic diffraction experiments from 2D crystals of protease-resistant PrP^Sc^ by cryo-electron microscopy (EM, [17]), molecular dynamics simulation [18] or electron tomography [19] all agree on the existence of an elementary brick of PrP^Sc^ subunits as core constituent. These subunits would be stacked by inter-subunit interactions to form the PrP^Sc^ assemblies (reviewed in [20,21,22]). The second part of the review reports on the identification of a PrP elementary brick from extractive PrP^Sc^ assemblies by methods coupling SV with unfolding/refolding process.

The third part of the review will discuss the implications of both prion heterogeneity and prion elementary brick on the current molecular aspects of the prion replication process. 

## 2. Strain-Dependent, Size-to-Infectivity Landscape of PrP^Sc^ Assemblies: Prions Are a Collection of Structurally Heterogeneous PrP^Sc^ Assemblies

A number of studies reported that prions are formed from a heterogeneous spectrum of PrP^Sc^ assemblies with respect to their tertiary and quaternary structure and biochemical properties [23,24,25,26,27,28,29,30,31,32]. However, the size-to-infectivity correlation was rarely defined [33]. The minimal size of prion infectious particles, which was estimated before the discovery of the prion protein [34,35,36], would correspond to two to four molecules of PrP^Sc^ [37]. 

In 2005, Silveira and Caughey published a seminal work on the identification of the most infectious prion particles, with respect to size. Using asymmetrical flow field-flow fractionation (A4F) to characterize individual PrP^Sc^ particles from 263K hamster prions, they showed that the most active protease-resistant particles with respect to PrP content, i.e., the particles conveying the strongest converting activity in robust-conversion assay and the highest specific infectivity by bioassay, were composed of multimers of 14–28 PrP-mers [24]. Oligomers smaller than a PrP pentamer were virtually devoid of any activity and large fibrils were less active. This work was amongst the first to address the relationship between PrP^Sc^ quaternary structure variations and infectivity under well-defined solubilization conditions.

The immediate question that followed Silveira’s work was as to whether the size of the most infectious prion particles varies with the strain type. Having identified solubilization conditions to separate the PrP^C^ from the PrP^Sc^ isoform by sedimentation velocity (SV) and sedimentation at the equilibrium (SE) protocols [23,25], we systemically compared the size distribution of PrP^Sc^ assemblies with that of infectivity amongst a panel of ovine and hamster cloned prion strains propagated in the ad hoc transgenic mice [23,25]. SV gradient analysis of brain at the terminal disease stage indicated that certain strains, categorized as fast (i.e., rapidly pathogenic for mice), contained a subset of oligomers of small size which was found to be the most infectious, whereas the PrP^Sc^ assemblies of larger size that mostly populated the brain were significantly less infectious. For the *slow* strains, the opposite situation was found. Here, the larger size multimers were the most infectious PrP^Sc^ assemblies [25]. The density profile of PrP^Sc^ assemblies tended to superimpose with that of the infectivity whatever the strain considered [23], meaning that differences in size, not shape (or association with lipids) were truly responsible for the observed differences amongst the assemblies and the strains in SV gradients.

**Figure 1:**
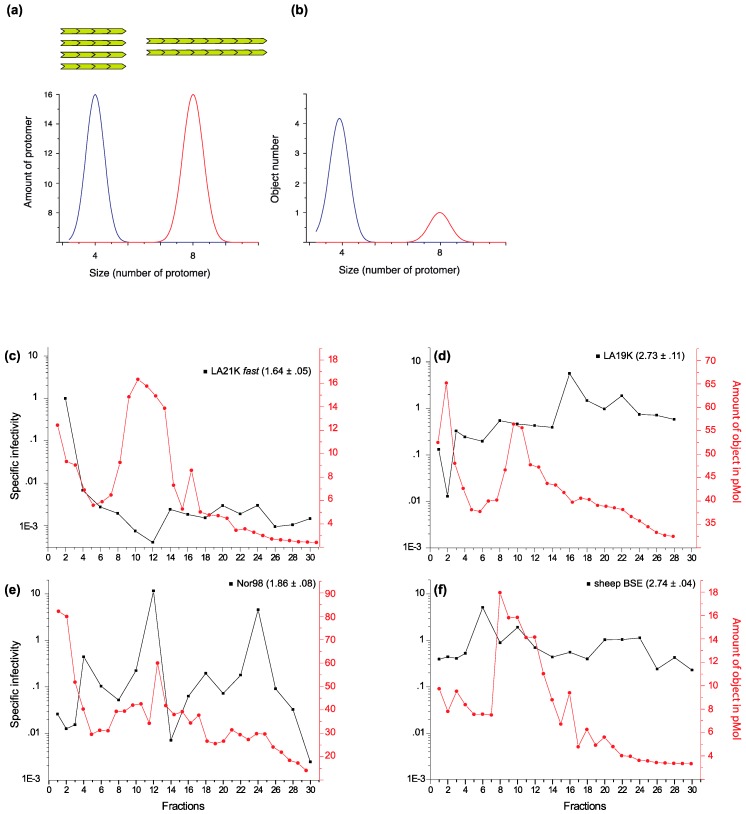
Size distribution of PrP^Sc^ assemblies and of their specific infectivity among different ovine prion strains. (**a**,**b**) Illustration describing the SV profile of two sets of assemblies (A and B) equivalent to the total number of protomers but different in terms of size. The sedimentogram is expressed as a function of protomer amount (**a**) or object number (**b**). The object number is also representative for the templating interfaces by assuming that templating can occur at least by one of the extremities. (**c**–**f**) SV profiles of ovine prion strains (original data from [25]). The specific infectivity of the SV-fractionated assemblies (black line) was calculated by dividing the relative infectivity of the assayed fraction by the relative amount of protease-resistant PrP^Sc^. The relative infectivity values were obtained from survival time bioassays in reporter tg338 mice. Specific infectivity values of (**c**) LA21K *fast* scrapie strain classified as *fast* strain (mean survival times of 56 days in tg338 mice), (**d**) LA19K (133 days), (**e**) Nor98 (186 days) and (**f**) sheep BSE (135 days), as *slow* strains. The amount of PrP^Sc^ assemblies in terms of object (red line) has been estimated by dividing the sedimentogram expressed in the equivalent of monomeric PrP by the theoretical fraction-molecular weight correspondence after calibration of the gradient for molecular weight [25]. The guanidine hydrochloride denaturation values of each strain are indicated ([Gdn]_1/2_ values in mol/L ± SEM, data from [23]).

The biochemical characteristics of one *fast* ovine strain termed LA21K *fast* were studied extensively. Bioassay indicated that the infectivity of the most infectious assemblies was not affected by increasing concentrations of proteinase K (PK). Their PK-resistance was overall slightly more pronounced compared to the larger-sized aggregates [23]. This implies that these assemblies, or at least the ones contributing to the infectivity, were not containing PK-sensitive species [26,27,30,31,38]. Treatment of the fractionated most infectious assemblies with enzymes known to preserve these PK-sensitive species further confirmed the absence thereof [25]. The absence of PK-sensitive PrP^Sc^ species contributing to infectivity allowed us to draw the specific infectivity of the SV fractions amongst the ovine strains, i.e., the amount of infectivity per number of PrP (Figure 1). Such graph was particularly informative with respect to the quaternary arrangements of the assemblies and the polymerization process.

According to canonical templating models, the PrP^C^ conversion and integration occurs at one or both extremities of PrP^Sc^ assemblies. For an equivalent PrP concentration (which is the readout of most size estimation methods) the number of replicative interfaces is an inverse of the assembly size (Figure 1a,b). Therefore, it is expected that the theoretical differences in the specific infectivity values between two assemblies differing by a factor of two in size would be low (and would decrease with size). As can be seen in Figure 1c–f, the specific infectivity of the protease-resistant PrP^Sc^ assemblies greatly varied within and between strains. For LA21K *fast* ovine strain, the small oligomers isolated in the top fractions exhibited values which were between 150- and 10,000-fold higher than all the other fractionated assemblies. In particular, the assemblies in fraction 12, where the bulk of PrP^Sc^ was isolated, exhibited 1000–10,000-fold reduced specific infectivity values (Figure 1c). Additionally, these fractions did barely differ in terms of objects (Figure 1c). For LA19K, the specific infectivity value peaked in fraction 18. Comparatively, the top fractions were 100 to 1000-fold less infectious (Figure 1d), despite being the richest in terms of objects. For Nor98, two types of assemblies exhibited the highest values, in fractions 12 and 24. The top fractions were 1000-fold less infectious (Figure 1e). For tg338-adapted BSE prions, the specific infectivity values did vary by more than 10-fold whatever the fraction studied (Figure 1f). Thus, there was a marked diversity in prion particle infectivity with respect to size and specific activity amongst the ovine strains. Given that the fractionated assemblies exhibited less than a ~10-fold range amplitude in terms of the number of objects, the differences observed in the specific infectivity values are particularly striking and point to profound differences in the PrP ultrastructural core amongst the assemblies rather than a variation of the number of replication interfaces.

The sedimentation studies at the equilibrium (SE) showed that the density values of infectivity and PrP^Sc^ tended to superimpose independent from the studied ovine strain (*fast* or *slow*) and therefore indicating that the pleiomorphic differences observed amongst the SV-fractionated assemblies were not linked to the architectural elements determining the density values [23]. Interestingly, these SE studies also indicated that the density of PrP^Sc^ assemblies exhibited relatively low values compared to proteins, including PrP^C^. PrP^Sc^ segregated in two peaks of 1.115 and 1.145 g/ml, while PrP^C^ had density values between 1.23 and 1.28 g/mL. These reduced density values suggested volumetric differences. Studies with recombinant PrP indicated that the alpha-helical to beta-sheet conversion has a profound effect on hydration and packing of the PrP protein [39,40,41,42], these two properties directly affecting the bulk density. 

The PrP solubilization conditions play an important role in the interpretation of PrP^Sc^ quaternary structure studies and PrP assemblies density estimation. Studies on the density/ aggregation size of prions are not new. Some were even conducted long before PrP was actually discovered (e.g., [43,44]). The solubilization conditions at that time were less or not controlled and thus the validity of the observations is rather uncertain. Some fractionation studies even employed crude, non-solubilized infected brain material [45]. The detergents that should be used must solubilize cellular membrane to a high degree without forming a micellar phase affecting PrP assemblies’ density and rheology. Glycosylphosphatidylinositol-anchored proteins associated with detergent-resistant microdomains such as PrP^C^ should be efficiently solubilized, and the activity of PrP^Sc^ in the detergent-solubilized state must be preserved. Correct solubilization of PrP^C^ is mandatory to avoid co-sedimentation of insoluble PrP^C^ particles with PrP^Sc^ [25]. In our study, we used a combination of *N*-Dodecyl β-D-maltoside and of *N*-Lauroylsarcosine sodium salt (sarkosyl) at relatively low concentration. Their combined use fulfills the criteria of solubilizing PrP^C^ to make it sedimenting as a monomeric protein [25] with expected protein density values [23]. To ensure that PrP^Sc^ sedimentation properties truly correlate with size or density, the solubilization conditions must ensure that the two PrP isoforms can be separated and sediment according to their aggregation state or density. For instance, sedimentation at the equilibrium with only sarkosyl as solubilizing detergent led to PrP^C^ floating in the top fractions of very low density, suggesting that PrP^C^ was still in detergent-rich microdomains, while PrP^Sc^ was found in the bottom fractions [46].

Collectively, the summarized data suggest that prion “most infectious particles” are greatly varying in size, in a strain-specific manner. Furthermore, fractionation studies in defined solubilization conditions demonstrate that prions are composed of a discontinuous collection of PrP^Sc^ assemblies with respect to specific infectivity values indicating different structures.

## 3. PrP^Sc^ Assemblies Are in a Constitutional Dynamic Equilibrium with Their Elementary Subunit

**Figure 2:**
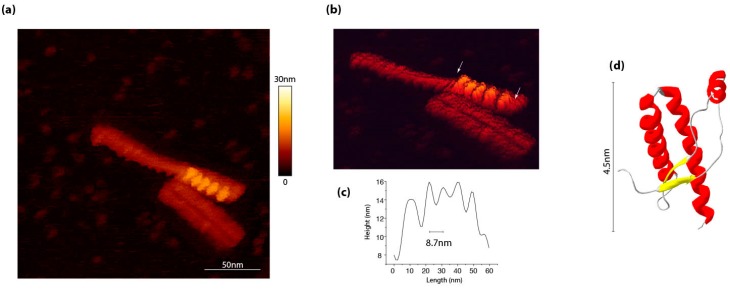
Motif repetition along PrP^Sc^ assemblies, as identified by atomic force microscopy. (**a**) 263K PrP^Sc^ assemblies purified according to a protocol by Wenborn et al. [52], as observed by atomic force microscopy in a liquid environment in sodium acetate buffer pH 5.0. (**b**) The scanning performed by using an Olympus AC406 nm cantilever in a QI mod revealed the existence of periodic element indicated between arrows in panel. (**c**) The axial distance between each periodical element is around 8.7 nm. (**d**) For comparison, the globular domain of ovine recombinant PrP (PDB:1TPX) has approximatively a diameter of 4.5 nm.

From a molecular viewpoint, the structural changes from the PrP^C^ native state to the pathogenic isoform are widely believed to occur through the structural adjustment of PrP^C^ monomers at the templating interface of PrP^Sc^ assemblies. As explained above, this current model, which is mostly derived from the Griffith and Caughey/Lansbury models [47,48] and from yeast prions (see §4.1 for more details) fails to explain the strain-specific differences in the specific infectivity amongst PrP^Sc^ subassemblies. Further, this polymerization model does not account for current PrP^Sc^ structural models, which all agree on the existence of a periodical repetition of a PrP oligomer subunit at the basis of the growing fibers [17,18,19,49,50,51]. Direct observations of PrP^Sc^ fibrils by atomic force microscopy allowed us to highlight a pattern repetition all along the assemblies. (Figure 2). This observation was fully consistent with previous work by tomography [19] and cryo-EM [49].

In the latter study, the authors observed different levels of repetition that they interpret as the superposition of sub-domains all along the protofilament. This repeated oligomer at the basis of PrP^Sc^ assemblies organization was for the first time isolated using a progressive, reversible denaturation process coupled to SV [53]. This subunit, termed suPrP^U^ (for sub-unit PrP trapped after urea treatment) was released after urea treatment of prion-infected brain homogenates. suPrP^U^ was PK-sensitive but highly stable, as its oligomeric structure was preserved up to 8M urea while PrP^Sc^ started disassembling at 1M urea. The existence of suPrP^U^ is a generic prion property, as this subassembly was isolated from hamster 263K prions and from two other unrelated strains termed T1^Ov^ and T2^Ov^ originating from the adaptation to ovine PrP transgenic mice of prions responsible for MM2-cortical forms of Creutzfeldt-Jakob disease [8].

suPrP^U^ from hamster 263K prions was poorly active with respect to templating activity and infectivity as compared to PrP^Sc^. However, it appeared to be highly dynamic. First, the structural rearrangements occurring during urea-induced disassembling were fully reversible. After urea removal by dialysis, suPrP^U^ refolded into assemblies termed rfPrP and the parameters defining the strain in terms of infectivity and disease phenotype were restored. This indicated that the strain structural determinants were enciphered into suPrP^U^. Further, the refolding of suPrP^U^ into PrP^Sc^ /rfPrP was under kinetical control, as the refolding was accelerated by suPrP^U^ concentration, without any removal of urea [53]. Second, PrP subunit release occurred not only in the urea-denaturing context but also in the absence of urea treatment by a simple high-speed dilution method. To distinguish between “physiological” and urea-induced release, these oligomers were termed suPrP. This method demonstrated that the suPrP release process was cooperative. Collectively, these data indicate that suPrP is in equilibrium with PrP^Sc^.

With regard to PrP^Sc^ structure, the above data suggest two different levels of organization with respect to stability. One structure would be highly sensitive to urea and responsible for packing of the subunits in PrP^Sc^ assemblies. The other would be highly stable and responsible for maintaining the oligomeric cohesion of suPrP. These two modes of packing may involve two distinct PrP domains, one for suPrP formation, the other for its condensation. Is such multiscale organization compatible with the models that have emerged as candidates for PrP^Sc^ structure in recent years? The most relevant ones are the parallel in-register β-structure [54,55,56] and two-, three- or four-rung β-solenoids [17,49,57,58], as they tend to address the constraints imposed by PrP^Sc^ post-translational modifications on its folding pattern. The multiscale organization does not support PrP^Sc^ assemblies being formed by the unique juxtaposition of parallel in-registered β-elements, where the interactions between each protomer are energetically equivalent [54,56]. Oppositely, the β-solenoids models show different types of interactions to form the fibril core and to stack the subunits. Structural analyses of glycosylphosphatidylinositol-anchorless PrP^Sc^ by cryo-electron microscopy suggest that these assemblies are formed from two fibrils, each being composed of dimers resulting from head-to-head and tail-to-tail contacts [49]. Given the lateral interactions between each fibril, one can suggest the elementary brick of these assemblies being formed by a dimeric complex of dimers or a tetramer, depending on the strength of the interactions. The size of suPrP^U^ was estimated by size exclusion chromatography [53] to correspond to a PrP trimer (±one monomer), or a mixture of dimers and tetramers. Such size would be consistent with most PrP^Sc^ structural models [19,49,51,57].

**Figure 3:**
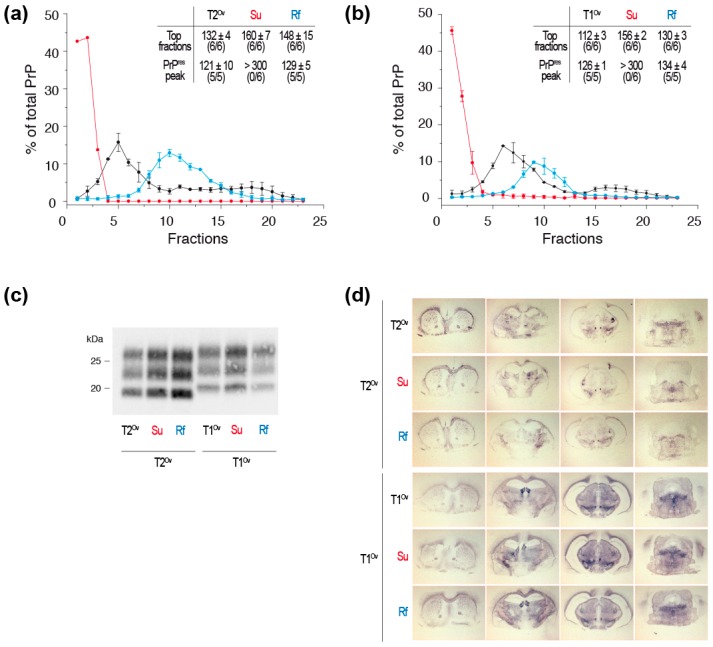
Infectivity and strain properties of suPrP^U^ and rfPrP from T1^Ov^ and T2^Ov^ prions. Tg338 brains homogenates were treated with 6M urea and either directly SV-fractionated to isolate suPrP^U^ oligomers or dialyzed before SV-fractionation to isolate rfPrP assemblies. The resulting fractions corresponding to suPrP^U^ and rfPrP were inoculated to groups of reporter tg338 mice (same method as in [53]). (**a**) Sedimentograms of untreated T2^Ov^ prions (black line), 6M urea treated T2^Ov^ prions (red line, suPrP^U^) and 6M urea-treated and dialyzed T2^Ov^ prions (blue line, rfPrP) (data from [53]) and as insert, incubation times of the mice inoculated with the top fractions (fractions 1–3) and the PK-resistant PrP^Sc^ (PrP^res^) peak (fractions 5–7 for untreated T2^Ov^, fractions 10–12 for suPrP^U^ and rfPrP). (**b**) Sedimentograms of untreated T1^Ov^ prions, 6M urea treated T1^Ov^ prions (suPrP^U^) and 6M urea-treated and dialyzed T1^Ov^ prions (rfPrP) (data from [53]) and as insert, incubation times of the mice inoculated with the top fractions (fractions 1–3) and the PrP^res^ peak (fractions 5–7 for untreated T1^Ov^, fractions 8–10 for suPrP^U^ and rfPrP). (**c**) PrP^res^ electrophoretic pattern and (**d**) PrP^res^ neuroanatomical deposition (histoblots) in the brains of tg338 mice inoculated with the top fractions and the PrP^res^ peak fractions from untreated T2^Ov^ and T1^Ov^ prions, 6M urea treated T2^Ov^ and T1^Ov^ prions (suPrP^U^) and 6M urea-treated and dialyzed T2^Ov^ and T1^Ov^ prions (rfPrP). The western blot and histoblot methods used here have been comprehensively described in [8,23,25,53].

Given the dynamic equilibrium between suPrP and PrP^Sc^ /rfPrP, the low infectivity/templating activity of the SV fractions corresponding to 263K suPrP^U^ might sound surprising. In titrations of PrP^Sc^ templating activity by cell-free assays, such as protein misfolding cyclic amplification (PMCA) [59,60], the samples are serially diluted. suPrP^U^ refolding rate may be so drastically reduced that the refolding may not occur during PMCA lapse of time (typically 48 h, [59,60]). In the animal bioassay, a similar outcome may occur due to clearance of inoculated material at the time of infection [61]. However, at low dilution (10^−1^), one out of five reporter mice developed the disease after inoculation of 263K suPrP^U^ [53], indeed suggesting a *slow* process. In the direct continuity of this work, we showed that the kinetic of suPrP^U^ to rfPrP refolding is strain-dependent. We performed bioassays of suPrP^U^ and rfPrP from the aforementioned T1^Ov^ and T2^Ov^ prions [8,53] in reporter ovine PrP transgenic mice (tg338 line) (Figure 3a,b).

The top fractions corresponding to suPrP^U^ were infectious for both T1^Ov^ and T2^Ov^ prions. They induced a 100% attack rate in the mice, yet with longer incubation times as compared to rfPrP or fractionated PrP^Sc^ assemblies. The use of standard dose-response curves ([8] and unpublished) indicated that the suPrP^U^ fractions from T1^Ov^ and T2^Ov^ were 100–1000-fold less infectious than rfPrP or PrP^Sc^ assemblies. As previously described with 263K prions [53], high infectivity titers were restored by rfPrP conditions. Mice inoculated with suPrP^U^ and rfPrP fractions showed similar PrP^res^ electrophoretic patterns and PrP^res^ regional deposition in the brain as compared to the untreated, fractionated parental inoculum (Figure 3c,d and [8]), further confirming that most aspects of the strain structural determinant are enciphered in the suPrP^U^ structure. Comparatively, these data indicate that the dynamic of $suPrP^{U}\to rfPrP$ refolding for T1^Ov^ and T2^Ov^ was much more efficient than for 263K prions [53]. This finding also suggests that $suPrP^{U}\to rfPrP$ refolding is strain-specific. 

Another interesting piece of information can be deduced from the observations reported in Figure 3 when comparing the relative infectivity levels (which are inversely correlated to the survival times) of the top fractions with those of the fractions containing the protease-resistant PrP^Sc^ (PrP^res^) peak, in untreated vs. refolded rfPrP material. After refolding of both T1^Ov^ and T2^Ov^ prions, the infectivity of the top fractions did not achieve the levels found in untreated material, while that of the PrP^res^ peak was fully restored. This suggests that the urea-induced disassembling and/or the refolding process has altered certain subassemblies with respect to their conformation, impacting their biological activity. This is particularly visible for T1^Ov^ prions. The “most” infectious particles, which segregated in the top fractions in the untreated brain material, were not recovered following the refolding process. Such assembly-dependent effect of the urea/refolding process further supports the view that the SV-fractionated assemblies are not a continuum of assemblies of different size with the same core structure.

Overall, correlating PrP^Sc^ quaternary structural transitions with prion biological activity during unfolding and refolding allowed revealing the existence of a mesoscopic organization in PrP^Sc^ through the packing of a highly stable oligomeric elementary subunit suPrP. Prion strain structural information is encoded in suPrP, reducing the minimal necessary structure size enharbouring the PrP^Sc^ structural information to a small oligomeric size. This approach also revealed the existence of an equilibrium between this elementary brick and PrP^Sc^ assemblies.

## 4. Time to Revisit the Molecular Basis of the Prion Replication Process?

Both the marked structural heterogeneity in PrP^Sc^ assemblies and the existence of suPrP raise perplexing questions on the fundamental principles of the prion replication, the prion spreading process and the prion biology. 

### 4.1. Prion Replication Process

The kinetic aspects of the prion replication process have been extensively described by quantitating infectivity or PrP^Sc^ levels in the brain [62,63]. Plotting prion accumulation as a function of time provides a typical sigmoidal shape, which has served to elaborate theoretical and mathematical models for prion replication. Among the most popular are the historical autocatalytic conversion model by Griffith (1967, [47]) followed, years later, by the nucleated-polymerization model (NPM) by Lansbury and Caughey (1995, [48]). In the NPM, each polymer is considered with the same conformation (n stacks of the infectious subunit) and specific activity (activity per PrP monomer). The marked differences observed in the specific infectivity values of SV-fractionated PrP^Sc^ assemblies relative to their “limited” variations of size (vide Supra) as well as PrP^Sc^ mesoscale organization based on suPrP elementary brick directly contradict a linear polymerization mechanism based on the addition and conversion of monomers of PrP^C^ at the extremities of the growing PrP^Sc^ aggregates. 

The questions then arise of how suPrP assembles de novo and serves as elementary brick during the replication process. In acquired prion diseases, the PrP^Sc^ infecting units may directly disassemble into suPrP due to a dilution effect induced by prion inoculum clearance [61]. At the resolution of our experiments, suPrP is a trimer ± one monomer [53]. A condensation process is required to generate infectious assemblies, meaning that the necessary and sufficient structural rearrangement could stem from a suPrP dimer (hexamer ± two protomers of PrP) for the replication process to proceed. Such dimers of suPrP trimers would constitute the minimal assembly size with replicative/infectious properties, a size consistent with that found by Silveira et al. with the same 263K hamster strain [24]. Alternatively, the infecting PrP^Sc^ templates may induce the formation of a trimer (±one monomer) by directly incorporating and converting three PrP^C^ monomers, or such trimer could exist as a minor assembly, in a displaced equilibrium with PrP^C^. In sporadic and genetic forms of the disease, the nucleus could be in equilibrium with trimers of PrP^C^, rather than with PrP^C^ alone as posited by the NPM.

How PrP^C^ is incorporated in the growing assemblies (i.e., the second level of organization in PrP^Sc^ assemblies) and the contribution of the dynamic equilibrium between suPrP and PrP^Sc^ to the replication process are tantalizing questions. 

### 4.2. Prion Propagon

The prion replication centers or propagons [64] refer to diffusive PrP assemblies harboring the prion strain structural information and are able to transmit it. Closely associated with these molecular entities, is their capacity to diffuse from cell-to-cell, or at distance, to ensure PrP^C^ conversion and in fine progression of the pathology. Despite their key role in the deadly prion progression, mammalian prion propagons have not been morphologically or structurally defined. It may also be expected that propagons morphotypes are strain-specific, given the disease heterogeneity, notably with respect to the disease incubation time. Both core mechanisms by Griffith and Lansbury/Caughey fail to describe how propagons are generated and recycled. The concept of active fragmentation mediated by housekeeping proteins such as heat shock proteins (HSPs) was introduced as critical underpinning for yeast prion spreading [65,66]. Mathematical modeling supported the importance of fibril breakage in the strain-specific replicative behavior of prions [67,68]. These concepts have been extrapolated to mammalian prions, despite the lack of relevant biochemical and biological evidence. According to both the Griffith and Lansbury/Caughey models replication process and the fragmentation hypothesis, the longest PrP^Sc^ polymers would generate the largest amount of propagons provided they are frangible. The rate of polymer fragmentation is believed to correlate with low conformational stability of PrP^Sc^ assemblies, as examined by their resistance to denaturation agents like guanidine hydrochloride or urea [69,70,71,72]. Yet, this correlation is not absolute and depends on the prion strain/host combination ([73] and Figure 1). 

We found that the subsets of small-sized assemblies were the most active in terms of templating activity and infectivity, especially in the *fast* ovine and hamster strains. Others observed that neuroinvasive strains were composed of assemblies which were either soluble or of small size, and reversely that poorly neuroinvasive strains were rather composed of large, fibrillar assemblies [23,25,26,28,74]. These observations collectively point to small, soluble or subfibrillar assemblies as propagons or at least as primary drivers of the disease tempo, either due to their higher templating activity or to their size allowing facilitated diffusion in the brain tissue. However, the data presented in Figure 3 suggest a more complex situation. The correlation between a *fast* disease pathogenesis and the subset of small size assemblies as the best replicator is not absolute. Indeed, T1^Ov^ and T2^Ov^ prions infect tg338 mice with relatively similar and short incubation periods [8]. However, the two strains differ in their most infectious particles with respect to size. For T1^Ov^, which has slightly longer incubation time than T2^Ov^, infectivity is associated with the subset of assemblies in the top fractions (fractions 1–3), as for the *fast* ovine and hamster strains. For T2^Ov^, infectivity is mostly associated with larger sized assemblies in fractions 5–7. The relationships between the PrP^Sc^ assembly heterogeneity and the disease tempo is thus complex. Multiplying the number and diversity of strains may allow uncovering fundamental principles.

The existence of suPrP, which is highly stable and in a dynamic equilibrium (i.e., in detailed balance) with PrP^Sc^ assemblies makes this elementary brick a relevant candidate for the prion propagon. Its small size compared to PrP^Sc^ assemblies makes suPrP highly diffusible. This hypothesis could explain the observations made by Chesebro et al. [75]. They demonstrated by microinjection of 22L prion strain in C57Bl6 and PrP knock-out mice that PrP^Sc^ aggregates were transported from the injection site to blood vessels by interstitial fluid flow within thirty minutes. They concluded that the rapid diffusion of such large assemblies (>500 kDa) can only be explained by a reduction of the size of the assemblies.

**Figure 4:**
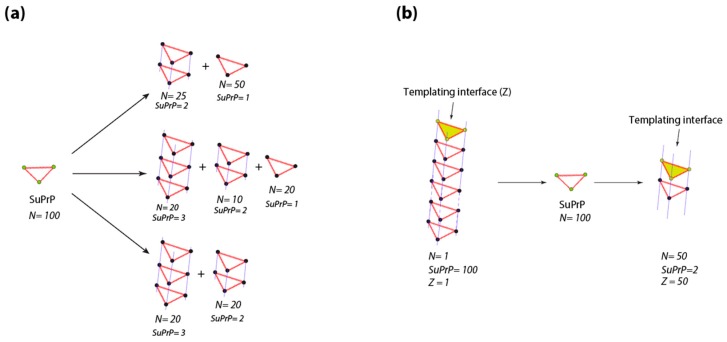
Relation between the number of templating interface and the detailed balance between PrP^Sc^ assemblies and suPrP. (**a**) According to the integer partition law and group representation theory ($p\left(N\right)=\frac{1}{4N\sqrt{3}}e^{\pi \sqrt{\frac{2N}{3}}}$, where N corresponds to the number of elements), 100 of molecules of suPrP can generate trough condensation 10^8^ possibility of PrP^Sc^ assemblies differing by size. As an illustration, three of them amongst the 10^8^ are presented. (**b**) A PrP^Sc^ assembly formed by the condensation of 100 suPrP presents only one templating interface (*Z* = 1). The existence of a detailed balance between PrP^Sc^ assemblies and suPrP makes that the PrP^Sc^ formed by 100 suPrP can rearrange into 50 PrP^Sc^ formed by the stacking of two suPrP, thus increasing the templating interface (*Z* = 50).

As emphasized above, the fragmentation process has been introduced in the prion field to describe the prion amplification step. Even if in fungus fragmentase proteins have been proposed to fragment PrP^SC^ (for review see [76]), in mammalian prions equivalent candidates and mechanisms fail. The detailed balance between PrP^Sc^ assemblies and suPrP makes the fragmentation process and the involvement of housekeeping machinery in the exponential increase of templating interface and spreading phenomenon dispensable. Indeed, the existence of an equilibrium between PrP^Sc^ and suPrP makes the number of templating interfaces per PrP^Sc^ assembly a dynamic parameter, depending on detailed balance between PrP^Sc^ assemblies and suPrP. As an example, with as little as 100 molecules of suPrP, theoretically, ~10^8^ possible ways of condensation exist, generating a plethora of assembly sizes and therefore templating interface. Figure 4a reports three ways of condensation of 100 molecules of suPrP among the 10^8^ possibility. Now, as shown in Figure 4b, if we take a PrP^Sc^ assembly formed by the condensation of 100 suPrP only one templating interface will be available (two, if both extremities of the assembly are able to template). Due to the existence of the detailed balance between PrP^Sc^ and suPrP, the PrP^Sc^ assembly initially formed by 100 suPrP molecules will generate suPrP which can condensate into a plethora of PrP^Sc^ assemblies with differing size. Among all the possibilities, one possible type of condensation corresponds to 50 molecules of PrP^Sc^ formed by the condensation of two suPrP, providing 50 templating interfaces (100, if the two extremities are involved). This specific example reflects that the existence of a detailed balance between PrP^Sc^ assemblies contributes to multiply the templating interface number without the participation in an energy consuming process such as fragmentation by housekeeping proteins. 

### 4.3. Prion Replication Process and Generation of PrP^Sc^ Assemblies Heterogeneity

As indicated before, the existing models of the prion polymerization process are unable to describe the existence and the generation of the structural diversification and heterogeneity within the brain at the molecular level. The concept of prion structural heterogeneity was first introduced to explain the emergence of new prion strain types during prion adaptation on cross-species transmission (comprehensively reviewed in [4,6]). One of the frequently advanced explanations is that prions are constituted of a cloud of substrains or a “quasi-species” [7] and when confronted with transmission barrier, the fittest substrain will emerge. Combinations of substrains in varying concentration have been found to co-replicate in the same brain, and their isolation and thus their phenotypic expression has been possible because of i) different tropism for the lymphoid tissue [8], ii) different capacity to adapt on cross-species transmission [77], iii) different capacities to accommodate different PrP^C^ levels [78] or PrP gene polymorphism [79]. Co-replication of structurally distinct assemblies such as those isolated by fractionation methods is more difficult to identify, as no obvious phenotypic differences have been noticed on the transmission of the isolated assemblies [24,25]. It may also be noted that the gold standard methods to type strains phenotypically may not be sufficiently discriminative to identify subtle pathological differences between structurally distinct assemblies from the same strain. Usually, these methods rely on measuring incubations times, quantitating the vacuolation score in brain-defined regions and observing PrP^Sc^ distribution in the brain, at the terminal stage of the disease. As a striking example, strain typing fails to differentiate cattle BSE prions from sheep-passaged BSE prions, except by differences in the survival times in multiple lines of reporter mice [80,81,82], as may be found with the SV-fractionated assemblies. These assemblies may also differ in structural elements independent of those dictating the strain phenotype in reporter animals. 

The maintenance of the PrP^Sc^ assembly structural heterogeneity all along the disease pathogenesis stands in apparent contradiction with the best replicator selection theory [83,84]. As the physicochemical properties of an assembly are dictated by its structure, two structurally distinct PrP^Sc^ subsets will exhibit two distinct replication dynamics and stabilities. More specifically they will compete for PrP^C^. However, this concept does not correspond to the experimental data on the structural heterogeneity of strains. Moreover, this structural heterogeneity of PrP^Sc^ assemblies is maintained on serial transmission in the same host [8,11,78].

What is the origin of the heterogeneity of PrP^Sc^ assemblies? It may be directly intrinsic to the prion replication process. The next question that immediately arises is whether these assemblies are generated independently or are they rather linked processes of co-generation or secondary diversification from a primary population of subassemblies (e.g., the first to be neosynthesized)? Further, based on the discussed data, have these subassemblies common or distinct suPrP? The host and the microenvironment may also be drivers of PrP^Sc^ heterogeneity. Due to the spatiotemporal diffusion of prion propagons in the brain, fluctuations in the infected host microenvironment may participate in PrP^Sc^ heterogeneity. This may include the diversity of prion-competent cells (e.g., astrocytes and neurons, [85,86,87,88]) populating specific brain areas, up or down-regulations of PrP^C^ levels [78], possibly due to response to infection [89], variations in PrP^C^ isoforms including glycoforms [90], all these factors being intertwined. 

It can be argued that the diversity of PrP^Sc^ assemblies identified by our SV-fractionation method was observed with prions passaged and cloned on mice overexpressing PrP. However, it must be noted that the strains that are compared were all propagated on the same transgenic mouse line, thus allowing direct comparison of their heterogeneity in PrP^Sc^ assemblies. Further, as published by Sandberg et al., most of the prion replication phase is not rate-limited by PrP^C^ expression levels, only the clinical onset would depend on PrP expression levels [13,14]. It remains entirely possible that certain structural polymorphs identified by the fractionation methods are specifically produced at the disease end-stage and are responsible for prion neurotoxicity. 

## 5. Conclusions

Both, the structural diversity of PrP^Sc^ assemblies and the discovery of suPrP oligomers as elementary bricks should stimulate new research to delineate the fundamental principles of the prion replication and spreading process. Recurrently in the prion literature, evidence arises concerning the coexistence of multiple conformers of PrP^Sc^ within prion strains or field isolates. Until now, the prion paradigm framework fails to mechanistically describe the coevolution of multiple sets of PrP^Sc^ assemblies. This review has pinpointed the potential importance of PrP^Sc^ assembly heterogeneity for prion biology, including strainness, propagation of prions in the brain and adaptation. How such heterogeneity participates in prion toxicity [13,14,91] remains an exciting field of investigation.
